# Role of the Epigenetic Modifier JMJD6 in Tumor Development and Regulation of Immune Response

**DOI:** 10.3389/fimmu.2022.859893

**Published:** 2022-03-14

**Authors:** Kai Wang, Chao Yang, Haibin Li, Xiaoyan Liu, Meiling Zheng, Zixue Xuan, Zhiqiang Mei, Haiyong Wang

**Affiliations:** ^1^ Research Center for Preclinical Medicine, Southwest Medical University, Luzhou, China; ^2^ National Engineering Research Center for Marine Aquaculture, Institute of Innovation and Application, Zhejiang Ocean University, Zhoushan, China; ^3^ Department of Pharmacy, 908th Hospital of Chinese PLA Joint Logistic Support Force, Yingtan, China; ^4^ Clinical Pharmacy Center, Department of Pharmacy, Zhejiang Provincial People’s Hospital, Affiliated People’s Hospital, Hangzhou Medical College, Hangzhou, China; ^5^ Department of Internal Medicine Oncology, Shandong Cancer Hospital and Institute, Shandong First Medical University and Shandong Academy of Medical Sciences, Jinan, China

**Keywords:** histone demethylation, JMJD6 inhibitor, epigenetic modification, immune response, tumor immunotherapy

## Abstract

JMJD6 is a member of the Jumonji (JMJC) domain family of histone demethylases that contributes to catalyzing the demethylation of H3R2me2 and/or H4R3me2 and regulating the expression of specific genes. JMJD6-mediated demethylation modifications are involved in the regulation of transcription, chromatin structure, epigenetics, and genome integrity. The abnormal expression of JMJD6 is associated with the occurrence and development of a variety of tumors, including breast carcinoma, lung carcinoma, colon carcinoma, glioma, prostate carcinoma, melanoma, liver carcinoma, etc. Besides, JMJD6 regulates the innate immune response and affects many biological functions, as well as may play key roles in the regulation of immune response in tumors. Given the importance of epigenetic function in tumors, targeting JMJD6 gene by modulating the role of immune components in tumorigenesis and its development will contribute to the development of a promising strategy for cancer therapy. In this article, we introduce the structure and biological activities of JMJD6, followed by summarizing its roles in tumorigenesis and tumor development. Importantly, we highlight the potential functions of JMJD6 in the regulation of tumor immune response, as well as the development of JMJD6 targeted small-molecule inhibitors for cancer therapy.

## Introduction

Posttranscriptional modifications of the N-terminal tail of histones, such as methylation, acetylation, phosphorylation and ubiquitination, play crucial roles in epigenetic regulation (1). Histone methylation is a key epigenetic factor that regulates transcription, chromatin status, and genome integrity, and participates in epigenetic regulation related disease processes. Methylation of histone arginine sites affects a variety of biological processes in the regulation of gene transcription, including cell signal transduction, DNA damage repair, cell development and cancer (2). Histone H3 and H4 are the two most modified histone proteins, and demethylation of histone H3 arginine 2 (H3R2me2) and histone H4 arginine 3 (H4R3me2) are key epigenetic events that frequently occur, including the regulation of chromatin dynamics, gene transcription, RNA shearing, etc (3). Generally, asymmetric H3R2me2 is associated with transcriptional inhibition, while its symmetric *di*-methylation or *mono*-methylation contributes to transcriptional activation (4); asymmetric H4R3me2 promotes transcriptional activation (5). Conversely, symmetric dimethylation triggers its transcriptional inhibition. Based on this, transcriptional activation and transcriptional inhibition may be related to the number and location of methyl groups on promoters and enhancers, as well as that of arginine guanidine groups. JMJD6, also known as KIAA0585, PTDSR, PTDSR1, is a member of the JMJC domain family of histone demethylases. It not only catalyzes the demethylation of H3R2me2 and/or H4R3me2 and regulates the expression of related genes (6), but also impacts a variety of non-histone modified factors such as HSP70, TNF receptor-associated factor 6, eIF3B, eIF3c, and G3BP1 (7, 8). Additionally, JMJD6 is also a histone targeting lysine hydroxylase, which contributes to lysine hydroxylation on distinct protein substrates for dynamic regulation of gene transcription (9). Multiple studies have shown that JMJD6 is conducive to the occurrence and development of a variety of tumors, including breast carcinoma, lung carcinoma, colon carcinoma, glioma, prostate carcinoma, melanoma, liver carcinoma, etc. Interestingly, it regulates some immune signaling pathways, such as Toll-like receptor related signal transduction, through its arginine demethylation. The relationship between the abnormal expression of this epigenetic gene in tumors and its immune regulation drives us to further explore the role of JMJD6 in tumorigenesis and tumor immunity. Herein, we review and update the latest role of JMJD6 in tumor development and regulation of innate immune response, providing a new understanding of the JMJC protein family as a potential target for tumor immunotherapy.

## Structure and Biological Function of JMJD6

**Figure 1:**
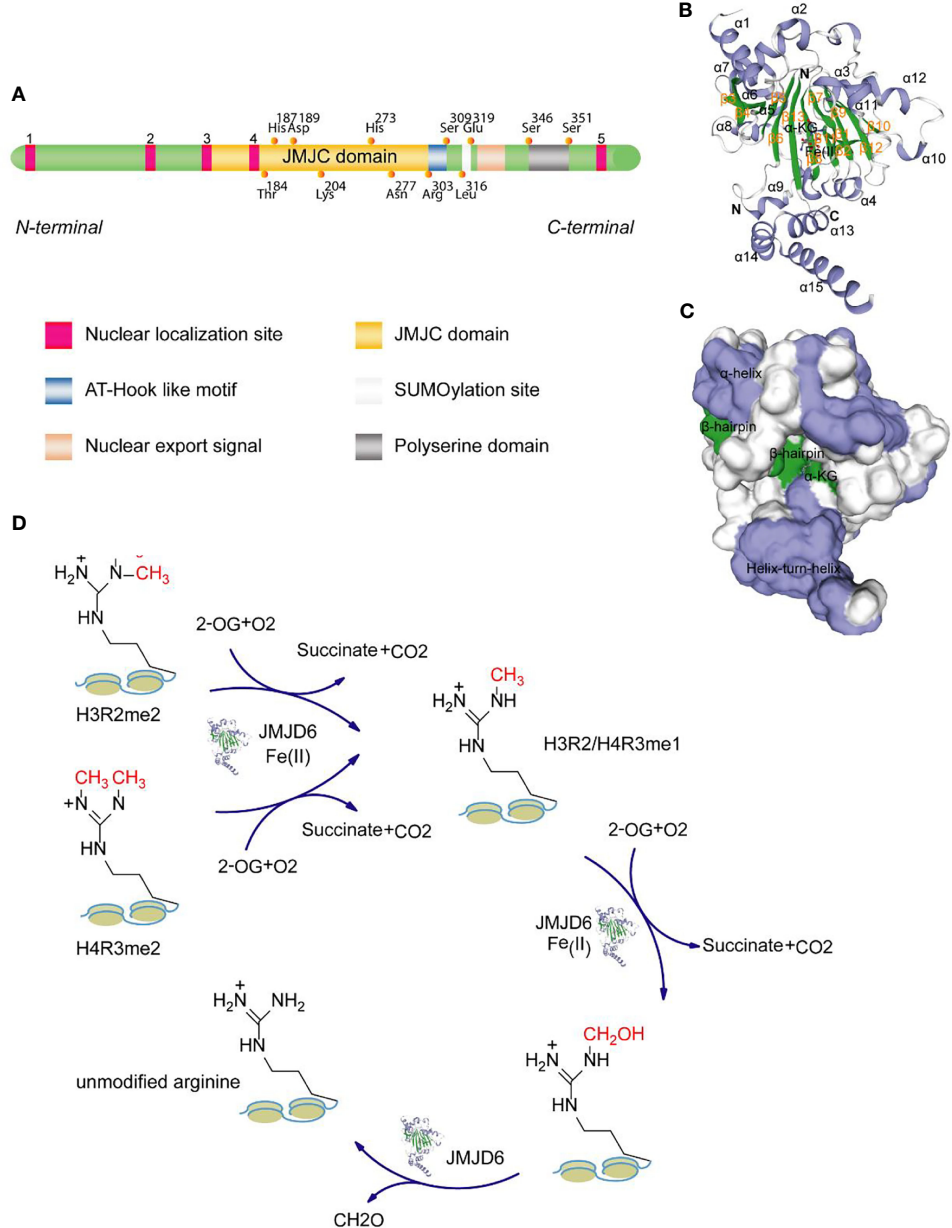
Domain structure of JMJD6 protein. **(A)** JMJD6 protein mainly consists of one JMJC domain, five nuclear localization sites, one AT-Hook domain, one presumed SUMOylation site, and one polyserine domain. **(B)** The stereo view of JMJD6 (PDB code: 6MEV) structure with α-KG and Fe (II) showing α-helix, β-hairpin, N-terminal domain and C-terminal domain. **(C)** The surface potentials of JMJD6 protein. The positively charged surface is grayish white, the negatively charged surface appears blue, and the surface of the neutral charge is green. The above two 3D views are drawn with the SWISS-MODEL software (11). **(D)** Schematic diagram showing the mechanisms for the demethylase activity of JMJD6.

JMJD6 is a 47-55kDa molecular weight member of the JMJC domain family. It is made up of 403 amino acids, and its genes are located on the 17qter chromosome (10). JMJD6 can be homologously dimerized by two α-helices at the N-terminus and C-terminus (amino acid residues at Glu^61^, Lys^68^, and Glu^322^, Glu^334^ of human JMJD6, respectively). It also forms trimers, tetramers, and pentamers based on the JMJC catalytic domain. Human JMJD6 contains one conserved JMJC domain, five nuclear-localization sites (NLS), one DNA binding domain (AT-hook like motif), one predicted SUMOylation site, and one polyserine domain (**Figure 1A**). JMJC domain (Pro^141^-Arg^305^) is the catalytic region of JMJD6 and forms double stranded -β helix (DSBH) fold of Fe (II)- and 2-OG-dependent dioxygenases (12). Five nuclear localization regions (Lys^6^-Arg^10^, Lys^91^-Arg^95^, Pro^141^-Lys^145^, Lys^167^-Pro^171^, and Arg^373^-Arg^378^) may facilitate the entry of functional proteins import into the nucleus (13). AT-Hook domain, located at Arg^303^ to Ser^309^ amino acid residues, mediates the binding of JMJD6 to DNA and/or RNA (14, 15). Although it has been found that there are positively charged regions in the grooves between the β-hairpin and helix-turn-helix of JMJD6 molecule (16) (**Figure 1C**), suggesting that JMJD6 can bind to nucleic acid. Whether this domain is directly involved in the binding of JMJD6 to RNA still needs further investigation. Potential SUMOylation site (Leu^316^-Arg^378^) may link to the protein stability and protein-protein interactions (17). The polyserine domain consists of amino acid residues at Ser^346^ to Ser^351^ and regulates nuclear or nucleolar shuttling of JMJD6 (18). The presence of the polyserine domain may promote JMJD6 interaction with serine/arginine-rich (SR)-like proteins with serine or arginine domains, i.e., some splicing factors (18). As shown in **Figure 1B**, the visual crystal structure of JMJD6 shows 15 α-helices and 13 β-hairpins, with the N-terminal bound by β13 and α9 sites and C-terminal bound by α13. In addition to β3 and β4, eleven of these structures exhibit eight antiparallel β chains, constituting the DSBH or cupin fold. The molecule Fe (II) binds to His^187^, Asp^189^ and His^273^ amino acid residues in the JMJC domain and determines the activity of the JMJD6 enzyme. Alpha-ketoglutarate (α-KG) binds to Fe (II) and Thr^184^, Lys^204^, and Asn^277^ residues in the central region.

The biological activity of JMJD6 is mainly driven by the JMJC central region (19, 20), which selectively catalyzes the demethylation of H3R2me2 and H4R3me2. The mechanism of demethylation is as follows: 1) In the presence of Fe (II) and 2-hydroxyglutaric acid, JMJD6 drives the *mono*-methylation and subsequent hydroxylation of arginine methyl groups on H3R2 and H4R3; 2) Subsequently, formaldehyde is produced through the formylation reaction, which facilitates the separation of methyl groups (**Figure 1D**). Demethylation is an important core epigenetic change that is involved in the regulation of transcription, chromatin structure, epigenetic and genomic integrity by specifically identifying chromatin active regions (21–23). A large number of studies have shown that JMJD6 is a key regulatory molecule in histone modification (such as diffusion of ubiquitination), transcriptional elongation and RNA splicing (14, 24) and that it is required for angiogenesis, cell differentiation and proliferation (25, 26). Based on the study of Hu et al., the proliferation of NIH3T3 fibroblast cells with JMJD6 knockdown was markedly reduced (27). However, loss of JMJD6 promotes rapid recovery of cell cycle checkpoints and improvement of cell survival rate after irradiation in distinct human cancer cell lines (28). We suggest that this seemingly contradictory biological effect may be related to cell types and status, irritation and duration.

## JMJD6 is Abnormally Expressed in Tumor

**Figure 2:**
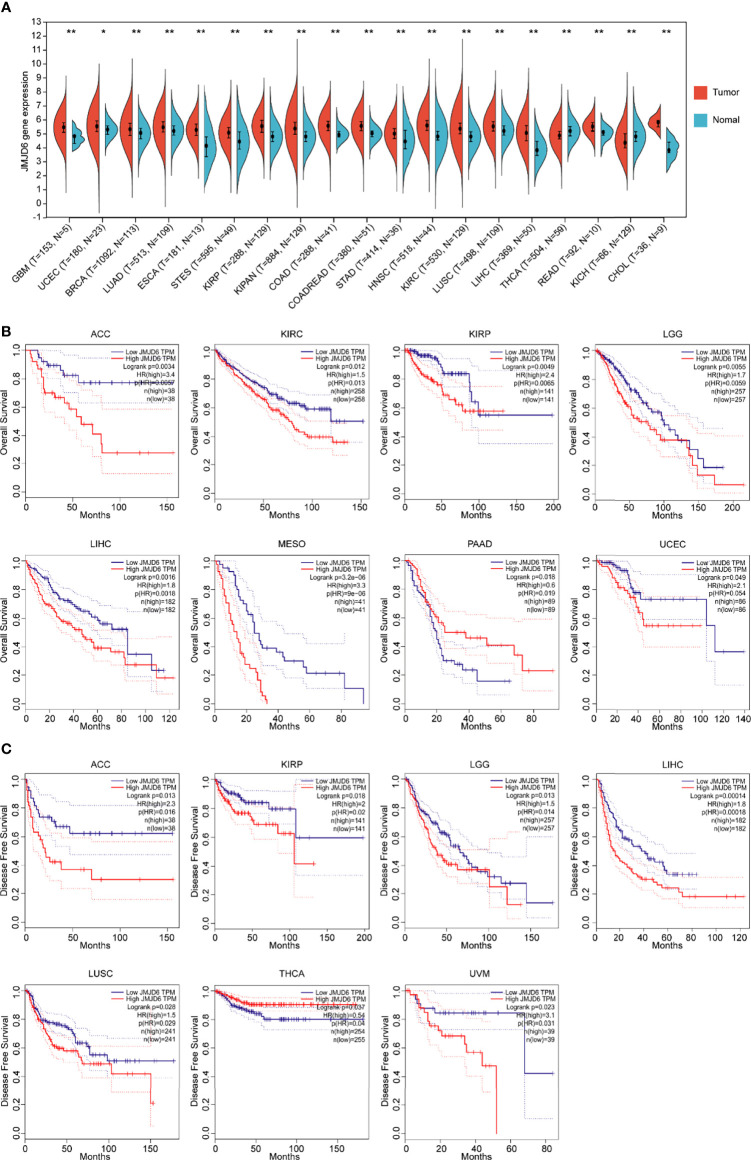
Bioinformatics analysis of JMJD6 in tumors. **(A)** JMJD6 expression in distinct tumor samples (T) and paired normal samples (N) based on TCGA. Log2 (TPM + 1) transformation was performed for each expression value and tumor types with sample numbers less than three were eliminated for differential analysis. *p < 0.05, **p < 0.01. **(B)** The correlation between JMJD6 expression level and OS; **(C)** The correlation between JMJD6 expression level and DFS. Correlation analysis was performed based on the GEPIA (http://gepia.cancer-pku.cn/) database.

The expression data of the JMJD6 gene in distinct human tumor samples were extracted from the Cancer Genome Atlas (TCGA) database. Then R software (Version 3.6.4) was used to calculate the difference in expression between human tumor tissues and the adjacent normal tissues. Significantly upregulated levels of JMJD6 gene were observed in 17 types of tumor samples, including glioblastoma multiforme (GBM), uterine corpus endometrial carcinoma (UCEC), breast invasive carcinoma (BRCA), lung adenocarcinoma (LUAD), esophageal carcinoma (ESCA), stomach and esophageal carcinoma (STES), kidney renal papillary cell carcinoma (KIRP), pan-kidney cohort (kidney chromophobe + kidney renal clear cell carcinoma + kidney renal papillary cell carcinoma) (KIPAN), colon adenocarcinoma (COAD), colon adenocarcinoma/rectum adenocarcinoma esophageal carcinoma (COADREAD), stomach adenocarcinoma (STAD), head and neck squamous cell carcinoma (HNSC), kidney renal clear cell carcinoma (KIRC), lung squamous cell carcinoma (LUSC), liver hepatocellular carcinoma (LIHC), rectum adenocarcinoma (READ), cholangiocarcinoma (CHOL) (**Figure 2A**). Further, Kaplan-Meier survival analysis was used to determine the correlation between JMJD6 expression levels and overall survival (OS) and disease-free survival (DFS). The bioinformatics tool Gene Expression Profiling Interactive Analysis (GEPIA) confirmed that patients with ACC, LGG, and LIHC showing increased JMJD6 mRNA levels had worse OS and DFS (**Figures 2B, C**). High levels of JMJD6 significantly reduced DFS in patients with KIRP, LUSC, and uveal melanoma (UVM), as well as OS in patients with KIRC and mesothelioma (MESO) (**Figures 2B, C**), suggesting the carcinogenic effect of JMJD6 in these tumors. However, JMJD6 expression was markedly diminished in several tumor types, i.e., thyroid cancer (THCA) and renal chromophobia (KICH), and its low expression resulted in poor DFS in THCA (**Figures 2A, C**).

**Figure 3:**
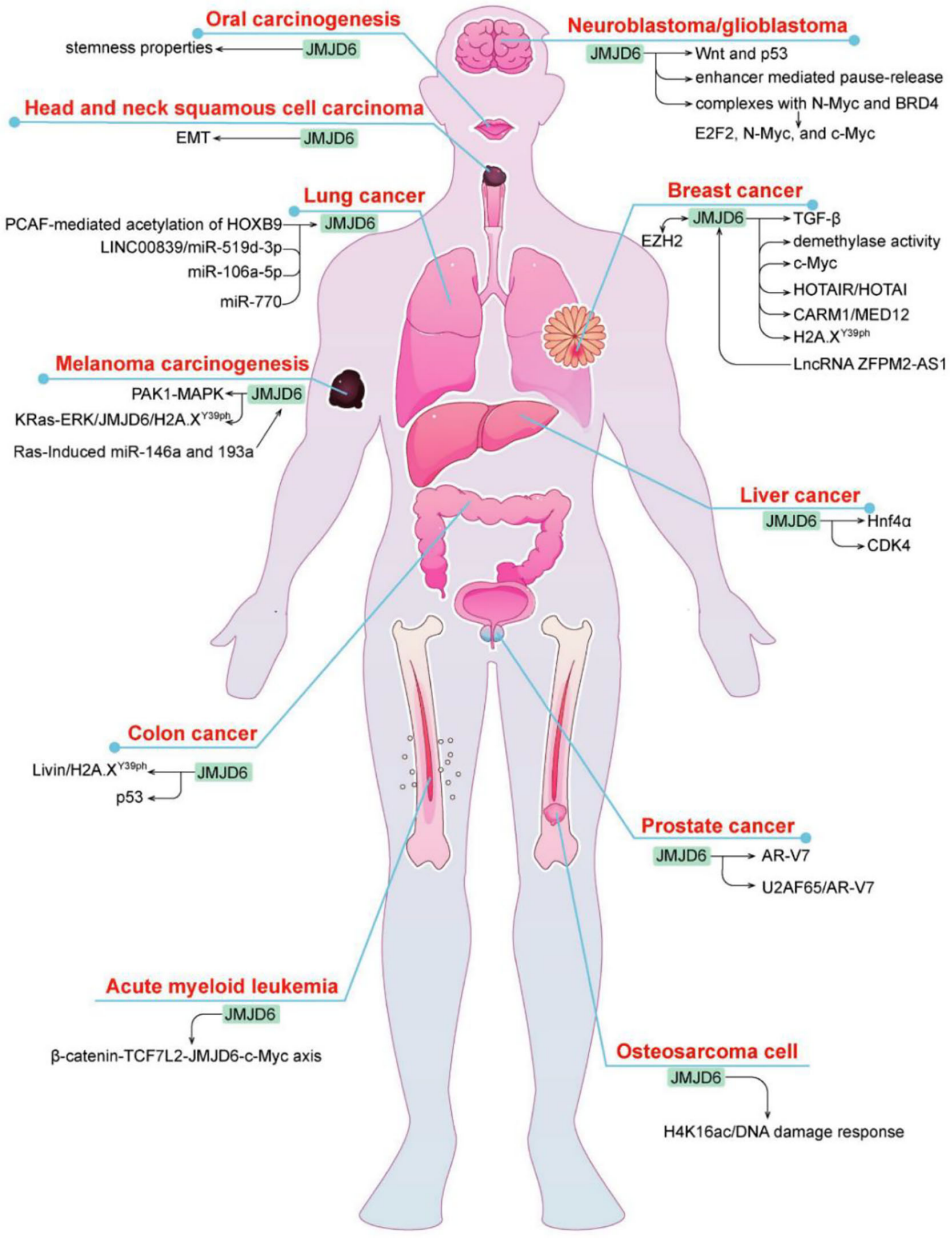
Multiple mechanisms of JMJD6 regulation in different tumors.

JMJD6 has a wide range of biological effects but its exact mechanism and clinical significance have not been fully determined. Recent studies revealed that JMJD6 is closely connected with cancer development and prognosis (29) (**Figure 3**). It is speculated that JMJD6 may be a novel biomarker and potential target for tumor prediction and treatment.

### JMJD6 in Breast Cancer

Breast cancer is a malignant tumor with the highest incidence in the world, which seriously affects women’s health. JMJD6 is one of the highly expressed genes in breast cancer and yet its role in breast cancer is two-sided. Poulard C et al. found that JMJD6 exhibited antitumor activity in MCF-7 breast cancer cell lines, while its high expression reduced DFS in patients with invasive breast cancer (30). JMJD6 is a downstream binding gene of LncRNA ZFPM2-AS1 and is involved in ZFPM2-AS1-mediated development of breast cancer by interacting with ZFPM2-AS1 (31). It was found that JMJD6 could bind to the p19ARF promoter and inhibited its mRNA and protein through H4R3me2a demethylation, thereby downregulating p53 levels and blocking c-Myc-induced apoptosis of breast cancer cells (32). Interestingly, JMJD6 co-overexpression with c-Myc synergistically triggered malignant phenotype, EMT and even metastasis in breast cancer as well as resulted in poor prognosis of ER+ breast cancer patients (32), suggesting it may be an important target of Myc-driven tumorigenesis and malignant progression of breast cancer. Zeste homologous enhancer 2 (EZH2) may be another chaperone co-regulating gene involved in JMJD6-mediated breast cancer progression. As a histone methyltransferase, EZH2 redirected HOX transcript antisense RNA (HOTAIR) to metastasis suppressor genes (i.e., PCHD5 and PCHD10) by directly binding to HOTAIR; JMJD6 positively regulated PCHD5 and PCHD10 and specifically targets HOTAIR (33). In breast cancer, JMJD6 and EZH2 were found to adjust the same cell cycle regulation genes and therapeutic targets through different mechanisms, resulting in poor prognosis, while inhibition of one of the two molecule does not prevent the malignant progression of cancer (33), which may be caused by the absence of regulatory regions of multiple cell cycle genes similar to those of JMJD6 in EZH2.

Estrogen is one of the key carcinogenic factors in hormone-sensitive cancers such as breast carcinoma. Data from some studies suggested that the role of JMJD6 in breast cancer depends upon ER status. In ER+/LN- breast cancer patients with endocrine monotherapy, it was found that the worse the clinical stage and differentiation of tumor and the prognosis of patients, the higher is the amount of JMJD6 protein; loss or knockdown of JMJD6 inhibited the progression of breast cancer *via* regulating the TGF-β pathway (34). On the one hand, JMJD6 regulated the recruitment of the MED12/CARM1 and thereby induced activation of ERα-dependent enhancer and coding gene. In this regard, JMJD6 is essential for estrogen/ERα-evoked cell growth and tumorigenesis of breast cancer (35). On the other hand, JMJD6 may be directly recruited to the HOTAIR promoter and then mediated HOTAIR independently of ER state (36). In addition, the mechanism of JMJD6 may differ in distinct subtypes of breast cancer. For example, JMJD6 not only had intrinsic tyrosine kinase activity but also phosphorylated Y39 of histone H2A.X (H2A.X^Y39ph^) in triple negative breast cancer (TNBC) cells, then autophagy regulated by JMJD6- H2A.X^Y39ph^ axis promoted cell growth (37).

### JMJD6 in Lung Cancer

JMJD6 is one of the most frequently altered genes in lung cancer, and plays a role in promoting lung cancer. In lung adenocarcinoma, the level of JMJD6 was remarkably increased, high JMJD6 expression was associated with size, grade, pTN status, pleural invasion, and poor prognosis (38). p300/CBP-associated factor-mediated the acetylation of HOXB9 can target JMJD6, leading to decreased cell migration and tumor growth (39). Recently, multiple studies have shown that JMJD6 was regulated by miRNAs (Mir-770, Mir-519D-3p, Mir-106a-5p) in lung cancer. For instance, JMJD6 was directly bound by Mir-770 targeting of its 3’-untranslated region (UTR), and acted as an oncogene in non-small cell lung cancer (NSCLC) cells, whereas overexpression of Mir-770 blocked tumor growth of NSCLC *in vitro* and *in vivo via* inhibiting JMJD6 and the WNT/β-catenin pathway (40). LINC00839 promoted the malignant development of lung cancer, because JMJD6 was directly targeted and inhibited by Mir-519D-3p, which was competitively sponged by LINC00839 (41). Similarly, another research showed that there was negative regulation between JMJD6 and Mir-106a-5p in cisplatin-resistant lung cancer cells, inhibition of JMJD6 reduced the proliferation, invasion and migration capacity, and thus alleviated the resistance of cisplatin (42).

### JMJD6 in Colon Cancer

Colon carcinoma is a common gastrointestinal malignancy, and its incidence has ranked the third among all gastrointestinal tumors. Although JMJD6 is highly expressed in colon tumors, its role in colon carcinoma is complex. For one thing, JMJD6 intercepted the transcriptional activity of p53 and increased its association with a p53 negative regulator MDMX by inhibiting p53 acetylation and promoting its hydroxylation (43), which may confer the pro-colon cancer activity of JMJD6. For another, JMJD6 facilitated the function of H2A.X^Y39ph^, which is a variant of histone H2A phosphorylated on serine39 (44). It can be degraded by an apoptosis inhibitor protein Livin and therefore controlled tumor initiation, whereas JMJD6 overexpression recovered the function of H2A.X^Y39ph^ and inhibited the advanced progression of colon cancer (44). The seemingly contradictory characteristics of JMJD6 may be owing to types of tumor tissue or cell lines and H2A serine phosphorylation site, yet further investigations are still needed.

### JMJD6 in Glioma

Glioma is the most common malignant primary brain tumor in adults. JMJD6 is up-regulated in about 80% of glioma tissues. The deletion of this gene leads to the proliferation, invasion and migration of glioma stem cells, and its molecular mechanism may be related to the down-regulation of Wnt pathway and up-regulation of p53 pathway (45). JMJD6 is also a key mediator that participates in the Myc pathway. A recent study showed that JMJD6 interacted with n-Myc and BRD4 to form protein complexes that induce the expression of E2F2 and Myc, which increased glioma carcinogenic risk. Inhibition of JMJD6 depressed the expression of E2F2, n-Myc and c-Myc, restrained cell proliferation *in vitro* and tumor growth *in vivo* and accelerated cell apoptosis (10). Moreover, enhancer-mediated transcriptional pause-release is also one of the reasons for JMJD6-induced tumorigenesis of glioma (46). These findings suggest that JMJD6 is a cancer-promoting gene and a novel promising target for anti-glioma therapy.

### JMJD6 in Prostate Cancer

As we know, endocrine resistance is a persistent problem in advanced prostate cancer that may be mediated by androgen receptor (AR)-V7. A phase II clinical trial showed that DNA-repair defects are more common in AR-V7-positive prostate cancer, which enables AR-V7-positive tumors more sensitive to immune checkpoint blockade (47, 48). Interestingly, JMJD6 was an essential regulator of AR-V7, and increased JMJD6 was correlated with higher levels of AR-V7, castration resistance and shorter survival, JMJD6 knockdown suppressed the growth of prostate cancer cells, AR-V7 levels, and the introduction of U2AF65 to AR pre-mRNA (49). Similarly, another study also verified that the JMJD6/U2AF65/AR-V7 axis may confer castration-resistant prostate cancer development (50). Thus, these results suggest that JMJD6 can be used as a therapeutic target for endocrine-resistant prostate cancer. Furthermore, the prognostic potential of JMJD6, especially in predicting recurrence, was confirmed by Cangiano M (51).

### JMJD6 in Melanoma

Liu X et al. reported that JMJD6 was significantly upregulated in melanoma, and the high expression of JMJD6 was related to poor prognosis and advanced clinicopathological stage of melanoma (52). JMJD6 functions as an oncogene that contributes to tumor progression by regulation of MAPK, Ras, and ERK signaling pathways. For instance, JMJD6 enhances MAPK signaling pathway by controlling PAK1 splicing and promotes melanoma cell proliferation, invasion and angiogenesis (52). Ras-induced mir-146a and mir-193a can target JMJD6 (53). Besides, Ras-ERK1/2 signaling stimulates the malignant progression of uveal melanoma *via* the regulation of JMJD6-mediated H2A.X^Y39ph^ (54).

### JMJD6 in Liver Cancer

JMJD6 may be involved in carcinogenesis and poor prognosis of liver cancer. A study showed that JMJD6 demethylated the hepatocyte nuclear factor 4 alpha (Hnf4α) promoter and inhibited its expression in the absence of PRMT1 (55), whereas Hnf4α is identified as a tumor suppressor and therapeutic target in liver cancer (56). JMJD6 also expedites the proliferation and controls the cell cycle of hepatocellular carcinoma cells by directly targeting CDK4 and modulating histone modifications on the CDK4 promoter (57).

### JMJD6 in Other Tumors

JMJD6 is highly expressed in oral squamous cell carcinoma (OSCC), head and neck squamous cell carcinoma (HNSCC), esophageal squamous cell carcinoma (ESCC), osteosarcoma, acute myeloid leukemia (AML), and renal cell carcinoma (RCC). JMJD6-IL4 axis may prompt changes in clinical phenotypes of OSCC cancer stem cells, JMJD6 accelerates self-renewal activity, migration/invasion, and drug resistance (58). High levels of JMJD6 urges the malignant progression of HNSCC by regulating epithelial mesenchymal transformation (59). Additionally, the identification of JMJD6 as a mutated target in ESCC will ultimately provide a potential predisposition variant and therapeutic opportunity (60). As a regulator of the DNA damage epigenome, JMJD6 can be recruited to DNA double strand breaks (DSBs) during microradiation *via* downregulating H4K16ac, and ultimately modulates the DNA damage response in radiation-induced osteosarcoma cells (28). JMJD6 promotes the proliferation of AML RN2 cell line and decreases the sensitivity of RN2 cells to various stresses (61). Besides, the β-catenin-TCF7L2-JMJD6-c-Myc axis plays an important role in BET protein inhibitors (BETi) resistance in AML cells. Knockout of JMJD6 reverses BETi-persister/resistance (BETi-P/R), while its overexpression causes BETi-P/R (62). In addition, ovarian cancer patients with high expression of JMJD6 have a poor prognosis (63). In RCC, JMJD6 transcription activation may contribute to tumor development, *via* inducing p300-mediated H3K27ac (29).

## Jmjd6 Regulates the Innate Immune Response

Increasing evidence suggests that epigenetic events and epigenetic genes are activated or suppressed in a variety of immune cells or immune responses, leading to the occurrence of different types of diseases (64, 65). Data from The Human Protein Atlas indicated that JMJD6 is highly expressed in endogenous immune cells. Earlier studies have shown that the activity of JMJD6 is related to intron retention and its release (66). JMJD6 can affect the level of medullary thymic epithelial cell (mTEC) mature proteins by inducing intron regulation of the Aire gene. The latter can express a variety of tissue-specific antigens and is regulated by Aire to establish self-immunity in the thymus (67). Deletion of JMJD6 results in reduced multi-organ autoimmunity in mice, decreased Aire protein expression, and increased disease progression.

Arginine methylation is a common post-translational modification that regulates a variety of functions, including cell cycle regulation of RNA processing and DNA replication. Meanwhile, arginine methylation affects gene transcription and splicing, and signal transduction, including some immune regulatory pathways (68). Tumor necrosis factor receptor associated factor 6 (TRAF6) is an E3 ubiquitin ligase. Under normal conditions, TRAF6 inhibits its ubiquitin ligase activity by methylation (69). Arginine demethylase JMJD6 can promote the demethylation of TRAF6, activation of NF-KB and production of LPS (70), suggesting the epigenetic gene JMJD6 is involved in the regulation of the endogenous immune pathway. Since TRAF6 is important for the transduction of endogenous immune signaling pathways downstream of most Toll-like receptors (TLRs). TLRs are expressed in a variety of cell types, including tumor cells, and can promote inflammation and immune responses. For this reason, exposure of TLR ligands regulated by arginine methylation of TRAF6-dependent TLRs pathway leads to the demethylation of TRAF6 by JMJD6.

**Figure 4:**
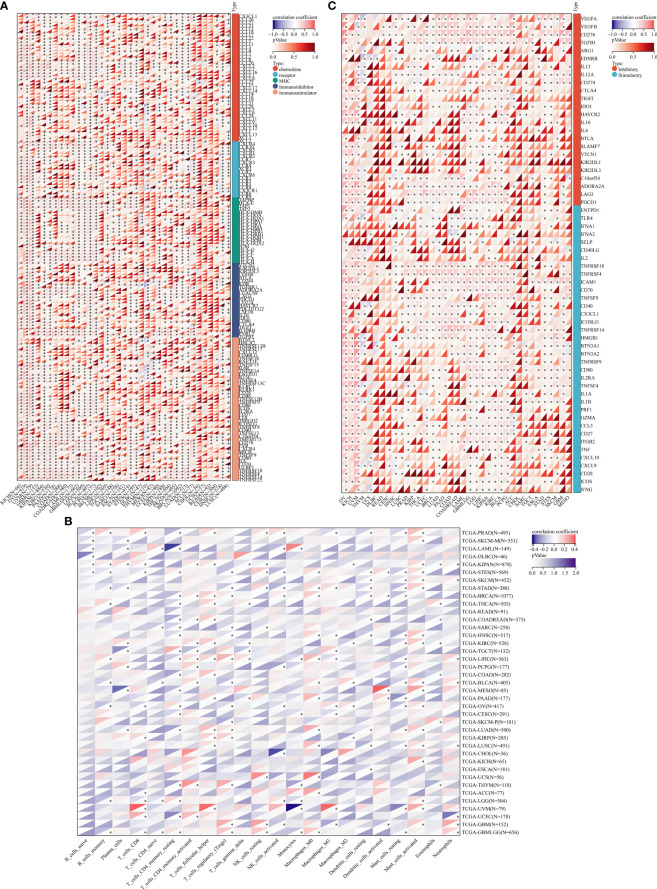
Bioinformatics analysis of the immunomodulatory role of JMJD6 in pan-cancer. **(A)** Correlation between JMJD6 and 150 immunomodulators (chemokines, receptors, MHCs, immunoinhibitors, and immunostimulators). **(B)** Correlation between JMJD6 and 22 tumor-related immune cells calculated with the CIBERSORT algorithm. **(C)** Correlation between JMJD6 and 60 genes associated with immune checkpoints. *P < 0.05.

JMJD6 catalyzes lysine hydroxylation and arginine demethylation of various substrates, such as splicing regulatory protein, transcription factors, and histones, in the Fe (II) and 2-OG-dependent manner (15, 71, 72). It regulates many biological functions including embryonic development, hematopoiesis, and nervous system but its role in the balance of immune response in tumors is still unclear. The tumor immune cycle reflects the anti-tumor immune response, including the release, presentation, initiation and activation of tumor cell antigens, infiltration of immune cells into tumor cells, recognition of tumor T cells, and killing of tumor cells by cytotoxic T lymphocytes (CTL) (73). The purpose of CTL is to kill tumor cells and boost the completion of tumor immune cycle. However, when antigen modification is on the surface of tumor cells, changes in recruiting inhibitory immune molecules and tumor microenvironment (TME), tumor cells can escape from CTL killing and induce immune escape. Recently, in a co-cultured experiment, JMJD6 deficiency enhanced the sensitivity of a variety of tumor cells to T cell killing (74), implying JMJD6 might be a CTL-evasion gene in tumor immunity. Further, the authors explored the role of JMJD6 interference in T cell killing of tumor cells. A Genome-wide CRISPR-CTL screening based on the Mouse Toronto KnockOut (mTKO) CRISPR Library revealed that antigen presentation mediated T cell toxicity was minimal in JMJD6-deficient tumor cells, making JMJD6-deficient tumor cells more suitable for CTL treatment. Pathway enrichment analysis showed that the Interferon Type 1 pathway was significantly enriched in JMJD6-deficient tumor cells. By analyzing tumor immune-related cytokines, they suggested that the combination of IFN and TNF-α cytokines may be the mediators of cell death in JMJD6-deficient tumor cells. Overall, JMJD6 may protect tumor cells against IFN and TNF-α cytokines-mediated cell death. To further understand the immunomodulatory role of JMJD6 in cancer, we downloaded the TCGA Pan-Cancer dataset (TCGA Pan-Cancer (PANCAN, N (TCGA sample number) =10535, G (ENSG ID number) =60499)) from the UCSC database (https://xenabrowser.net/)), and extracted the expression data of JMJD6 and 150 immunoregulatory genes, 60 immune checkpoint-related genes as well as 22 tumor-associated immune cells in pan-cancers. Bioinformatics analyses aimed at describing the immune role of JMJD6 are pivotal in determining different types of cancers that might benefit from the anti-JMJD6 immunotherapy. As we all know, TME is a very complex environmental system related to tumor cell survival, including immune cells, fibroblasts, vascular endothelial cells, extracellular matrix and various signaling molecules. It promotes the expression of some oncogenes and inhibits immune cells from performing their immune functions. Bioinformatics analysis results uncovered that JMJD6 was negatively associated with a series of immunoregulatory genes (i.e., CCXL2, CCL22, CCL25, CCL14, CCL16, CXCL9-12, etc) in LUSC, HNSC, UCS, MESO, THYM, DLBC, UCEC, LIHC, and SARC (**Figure 4A**), and was negatively correlated with most tumor-infiltrating immune cells (i.e., plasma cells, CD8+ T cells, resting memory CD4+ T cells, gamma delta T cells, activated NK cells, monocytes, M1 macrophages, and dendritic cells) in pan-cancer (**Figure 4B**). The infiltration of those immune cell subtypes can activate the anti-tumor immune response by inducing humoral or cellular immunity, and reshapes the immune system and inhibiting the TME. However, JMJD6 was found to be positively correlated with regulatory T cells (Tregs) that inhibit anti-tumor immunity and M2 macrophages that promote tumor growth (**Figure 4B**). In conclusion, the expression pattern of JMJD6 may be inflamed TME-specific. In other words, JMJD6 may maintain the balance of tumor microenvironment and tumor cell growth by selectively regulating various immune cells and immunomodulatory genes that have pro-cancer or anti-cancer effects. Furthermore, the expression of JMJD6 is mutually exclusive of the tumor immune checkpoints, such as VEGFA, ARG1, EDNRB, IL13, IL12A, CD274 and KIR2DL3 in the majority of cancers, like THYM and HNSC, etc (**Figure 4C**). We believe that JMJD6 has great potential as a novel target for cancer immunotherapy. However, how JMJD6 regulates epigenetic events and immune responses to affect the progression of malignant tumors still needs further research.

As an epigenetic factor, the role of JMJD6 in tumors has been proved to be related to the regulation of some crucial target molecules, including p53, Wnt, MAPK, Ras, ERK, TGF-β, CARM1, c-Myc, CDK4, ZFPM2-AS1, AR-V7, estrogen signal, etc. These targets modulate immune responses in TME on different levels. For example, p53 is closely related to the function and immune response of the body’s immune system (75). On the one hand, abnormal expression of p53 functions as a transcription factor that directly activates the expression of relevant genes in immune cells, thus regulating the proliferation of tumor cells; On the other hand, it can be used as tumor-associated antigen to stimulate an immune response. Studies have shown that abnormal expression of the Wnt signaling may also disrupt tumor immunodetection and promote tumor immune evasion (76). MAPK signal transduction inhibits the activity of immune cells in the TME by upregulating the release of cytokines such as IL-6 and IL-10 (77). Ras drives immune checkpoint molecules on the surface of tumor cells to bind to homologous receptors on immune cells such as CD4+ T cells, CD8+ T cells and NK cells, thereby reducing the killing ability of cancer cells; it also leads to increased levels of PD-L1 and B7-H3 and promotes immune escape of tumor cells (78). ERK activation triggers the invasion of specific myeloid cell subsets and the formation of TME (79). Advanced tumor cells can secrete a large amount of TGF-β, and high levels of TGF-β enable immature T cells to transform into Tregs, thus obstructing antigen presentation and leading to immune escape of advanced tumor cells (80). In the tumor microenvironment, the inactivation of CARM1 can activate the DNA damage response of tumor cells, improve the TME, and promote the aggregation of innate immune cells (81). In addition, inhibition of its activity also enhances the activity of killer T cells and stimulates T-cell-mediated anti-tumor immunity (81). C-Myc induces tumor cells to evade immune detection and cytotoxic T cell responses by decreasing antigen presentation of HLA-DM and CD4+ T cells and down-regulating the expression of adhesion molecules (LFA-1, CD54, CD58) and costimulatory molecules (i.e., CD40) (82). CDK4 is an important protein regulating cell cycle and affects immune escape and tolerance of tumor cells (83). ZFPM2-AS1 may regulate the tumor immune microenvironment by influencing cytokine/cytokine receptor signaling pathways and IL-2/signal transduction factors and transcriptional activator 5 (84). A phase II clinical trial shows that DNA-repair defects are more common in AR-V7-positive prostate cancer, which may make AR-V7-positive tumors more sensitive to immune checkpoint blockade (47, 48). Estrogen/ERα signaling promotes the accumulation of myeloid suppressor cells (MDSCs), the latter of which are immune cells associated with tumor resistance (85). Based on the above discussion, it is speculated that JMJD6 may be involved in carcinogenesis through the tumor immune response mediated by these key target molecules.

## JMJD6 Inhibitors

**Figure 5:**
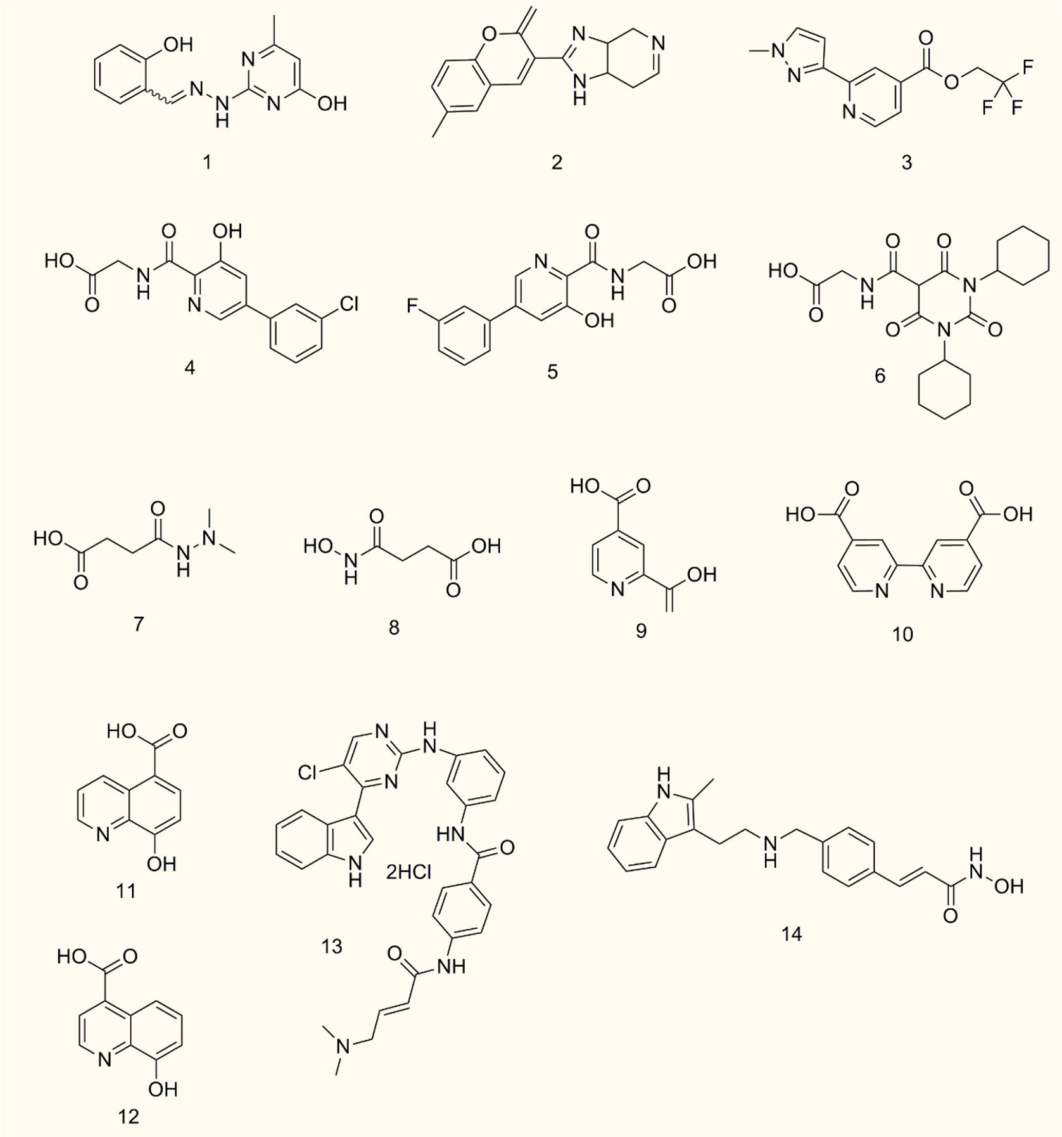
Chemical structures of representative JMJD6 inhibitors.

JMJD6 is a dioxygenase dependent on 2-OG and Fe (II). Its central JMJC domain has specific catalytic sites that target 2-OG/Fe (II) and regulate transcription and splicing by catalyzing the demethylation of histone/non-histone arginine or lysine hydroxylation (19, 20). Therefore, JMJD6 inhibitors targeting the catalytic sites may be effective strategies to interfere with its carcinogenesis. Owing to the crucial roles of JMJD6 in different cancers, inhibitors of JMJD6 may have potential antitumor effects. To date, only a few JMJD6 inhibitors have been identified, and no JMJD6 inhibitor has been applied clinically. This is probably due to the low toxicity and low selectivity of most JMJD6 inhibitors. Thus, selective structural modification and polymer chemistry technology can be used to enhance anti-tumor efficacy and improve the drug selectivity of tumor cells. Meanwhile, the classification of different tumor patients and the selection of appropriate JMJD6 inhibitors for individualized treatment will also be the trend of anti-tumor therapy. Based on the immunomodulatory effect of JMJD6, combination therapy, such as JMJD6 inhibitors combined with immunomodulators, can also maximize the treatment efficiency of cancer patients and reduce drug resistance. Currently, we summarize the development of JMJD6 inhibitors (**Figure 5** and **Table 1**).

**Table 1:** Inhibitory activities of representative JMJD6 inhibitors.

Compound Number	Chemical name or compound name	IC50 or Kd value
1	SKLB325	0.78 μM
2	WL12	0.22 μM
3	Compound 7p	0.68 μM
4	Vadadustat	7 ± 1.60 (14 ± 5.50) μM
5	AKB-6899	9 ± 1.5 (3 ± 2.80) μM
6	GSK1278863/Daprodustat	(10 ± 1.20) μM
7	Daminozide	94 μM
8	Structurally related succinyl hydroxamic acid derivative 3	25 μM
9	2,4 PDCA	13 ± 1.30 (6 ± 1.30) μM
10	2,4 BPDCA	6 ± 1.60 (7 ± 1.80) μM
11	IOX1	(10 ± 1.50) μM
12	8-hydroxyquinoline derivative 2	(5 ± 1.30) μM
13	THZ1	N.D.
14	panobinostat	N.D.

N.D. stands for not detected.

SKLB325, showing an IC_50_ value of 0.78 μM against JMJD6, was firstly reported by Zheng et al. (63). It was found that SKLB325 significantly inhibited the proliferation of SKOV3 cells, markedly decreased the weight of tumors, and prolonged the survival time of tumor-bearing mice (63). In addition, SKLB325 not only inhibited JMJD6-mediated carcinogenesis of RCC but also makes RCC sensitive to sunitinib (29). Subsequently, Ran T et al. calculated and evaluated the drug sensitivity of JMJD6 through the structure of the JMJC domain, and developed a silicon protocol for virtual screening, to screen small molecule inhibitors of JMJD6. Finally, based on experiments such as inhibition of JMJD6 demethylation activity and suppression of JMJD6 proliferation in WL12 tumor cells, with an IC_50_ value of 0.22 μM, was identified to be a specific small molecule inhibitor of JMJD6 (20). Recently, Wang TQ et al. used a series of studies, including molecular docking, deep structural optimization, and correlation analysis between structure and activity, to discover novel JMJD6 inhibitors, then found a new compound 7p (IC_50_: 0.681 μM), which has good selectivity (>100 fold) (19). However, the role of compound 7p in tumor inhibition was not explored.

Except for SKLB325, WL12, and compound 7p, the other four HIF prolyl hydroxylase inhibitors (Vadadustat, AKB-6899, GSK1278863/daprodustat, and panobinostat) may be potential JMJD6 inhibitors (86) for cancer therapy. For example, Vadadustat and GSK1278863/daprodustat reconstructed tumor vessels and improved TME to inhibit tumor growth (87). Additionally, the antitumor effect of AKB-6899 was also reported, AKB-6899 reduced tumor growth of melanoma and suppressed angiogenesis *via* increasing sVEGFR-1 production of tumor-associated or GM-CSF treated macrophages (88).

Furthermore, it also was reported that 2,4 PDCA, 2,4 BPDCA, Daminozide, the structurally related succinyl hydroxamic acid derivative 3, IOX1, and 8-hydroxyquinoline derivative 2, maybe potential inhibitors of JMJD6 (10). Among them, anti-tumor effects of IOX1 in colorectal cancer (89) and chemo-immunotherapy (90) have been found.

Co-inhibition of THZ1 (the CDK7/super-enhancer inhibitor) and panobinostat (the histone deacetylase inhibitor) could synergistically suppress JMJD6, resulting in increased cell apoptosis *in vitro* and tumor inhibition in mice with neuroblastoma. These findings suggest that THZ1 and panobinostat are nonspecific inhibitors of JMJD6 (32).

## Conclusion and Prospect

**Figure 6:**
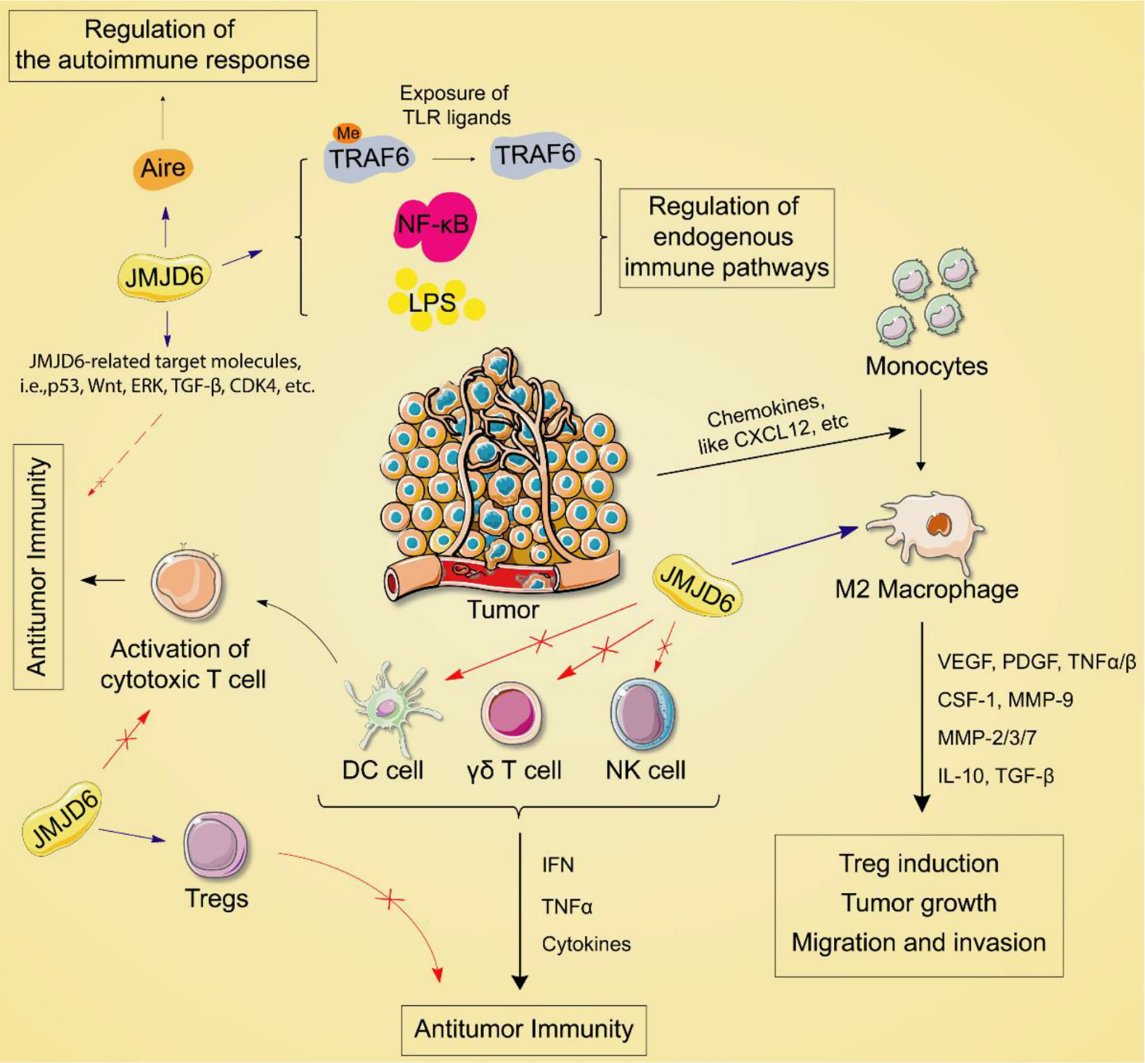
Potential immunomodulatory mechanisms of JMJD6.

In summary, JMJD6 is involved in transcriptional chromatin structural epigenetic and genomic integrity regulation through selective demethylation. A large number of studies have shown that JMJD6 expression is increased in many tumors and contributes to the development and progression of tumors. In this article, we have discussed the biological role of JMJD6 and its important role in tumorigenesis, as well as immune response (**Figure 6**), and found JMJD6 is likely to become an attractive target for novel tumor immunotherapy and prevention. However, how JMJD6 regulates immune inflammatory factors through epigenetic modification to affect tumor development, and whether JMJD6-mediated arginine demethylation and lysine hydroxylation affect tumor immunotherapy are unclear. Therefore, studying the key roles and mechanisms of JMJD6 in tumor treatment will provide a sufficient experimental and theoretical basis for future clinical applications. In addition, more and more 3D crystal structures or domains associated with JMJD6 will be revealed in the future, which may be more conducive to the rational structure and highly selective drug design of JMJD6 inhibitors.

## Author Contributions

KW drafted the manuscript and drew the original artwork. KW, CY, ZX, ZM, and HW revised the manuscript. HL, XL, and MZ edited the manuscript and contributed to the systematic evaluation of articles and literature. All authors made substantial contributions to the manuscript and approved the submitted version.

## Funding

This work was supported by Luzhou Science and Technology Bureau (2021-JYJ-55), the Research Fund of Southwest Medical University (41/00190026; 2021ZKQN108), and the Start-Up Research Funding of Southwest Medical University (41/00040179).

## Conflict of Interest

The authors declare that the research was conducted in the absence of any commercial or financial relationships that could be construed as a potential conflict of interest.

## Publisher’s Note

All claims expressed in this article are solely those of the authors and do not necessarily represent those of their affiliated organizations, or those of the publisher, the editors and the reviewers. Any product that may be evaluated in this article, or claim that may be made by its manufacturer, is not guaranteed or endorsed by the publisher.
